# Dissociable Effects of Reward on Attentional Learning: From Passive Associations to Active Monitoring

**DOI:** 10.1371/journal.pone.0019460

**Published:** 2011-04-29

**Authors:** Chiara Della Libera, Andrea Perlato, Leonardo Chelazzi

**Affiliations:** 1 Department of Neurological, Neuropsychological, Morphological and Movement Sciences – Section of Physiology and Psychology, University of Verona, Verona, Italy; 2 National Institute of Neuroscience, Verona, Italy; Ecole Polytechnique Federale de Lausanne, Switzerland

## Abstract

Visual selective attention (VSA) is the cognitive function that regulates ongoing processing of retinal input in order for selected representations to gain privileged access to perceptual awareness and guide behavior, facilitating analysis of currently relevant information while suppressing the less relevant input. Recent findings indicate that the deployment of VSA is shaped according to past outcomes. Targets whose selection has led to rewarding outcomes become relatively easier to select in the future, and distracters that have been ignored with higher gains are more easily discarded. Although outcomes (monetary rewards) were completely predetermined in our prior studies, participants were told that higher rewards would follow more efficient responses. In a new experiment we have eliminated the illusory link between performance and outcomes by informing subjects that rewards were randomly assigned. This trivial yet crucial manipulation led to strikingly different results. Items that were associated more frequently with higher gains became more difficult to ignore, regardless of the role (target or distracter) they played when differential rewards were delivered. Therefore, VSA is shaped by two distinct reward-related learning mechanisms: one requiring active monitoring of performance and outcome, and a second one detecting the sheer association between objects in the environment (whether attended or ignored) and the more-or-less rewarding events that accompany them.

## Introduction

It is well established that the amount of information that can be fully processed at any given moment is limited. Considering for instance the richness of particulars offered by a typical visual scene in front of us, it is easy to verify that we cannot be aware of all its details at all times. Concurrently present stimuli compete with each other to gain access to limited processing resources, and visual selective attention (VSA) is the cognitive process responsible for supervising and resolving this competition. Hence, only a limited amount of the total incoming information – deemed as relevant – has a role in behavioral guidance. VSA resolves stimulus competition by means of a twofold mechanism. On the one hand it enhances the perceptual saliency of objects which are relevant for the current behavioral goals; on the other it suppresses the saliency of the less relevant, and possibly distracting items, whose processing may in fact harm the execution of the intended behavior [1]–[4].

In recent years it has become evident that VSA does not operate in a rigid fashion, but can instead be adjusted to the given situation, maximizing the efficiency of behavior. A vast literature has shown that attentional processes are subject to *learning*, becoming more efficient in familiar environments, and for frequently encountered stimuli, suggesting a strong interplay between VSA and memory mechanisms (e.g., [5]–[9]). In visual search tasks – where subjects must report the presence of a given target within an array of distracting items – such learning effects have been observed both in the form of speeded detection of more frequent target stimuli and powerful suppression of distracters, in all cases leading to more efficient processing of the practiced stimulus arrays [10]–[25]. Recordings of single unit activity from the inferotemporal cortex of behaving macaques suggest that the enhanced behavioral relevance of frequently attended stimuli derives from significant changes in their representation at the neural level: following extensive training, neuronal responses are increased for items repeatedly shown as targets, while they are reduced for those consistently displayed as distracters [26].

**Figure 1:**
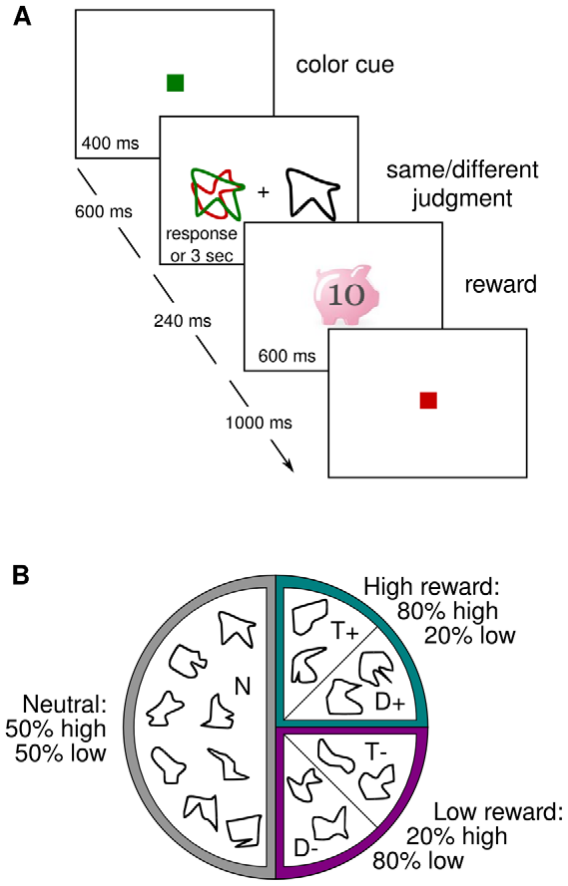
Training task and reward schedule. a) Subjects performed a same/different judgment between one of two overlapping shapes on the left (designated by the color of a central cue displayed prior to each stimulus display) and a single shape presented on the right. During training each correct response was followed by a reward signal indicating a high or low monetary win. b) Arrangement of stimulus shapes into biased reward categories for one exemplar subject.

**Figure 2:**
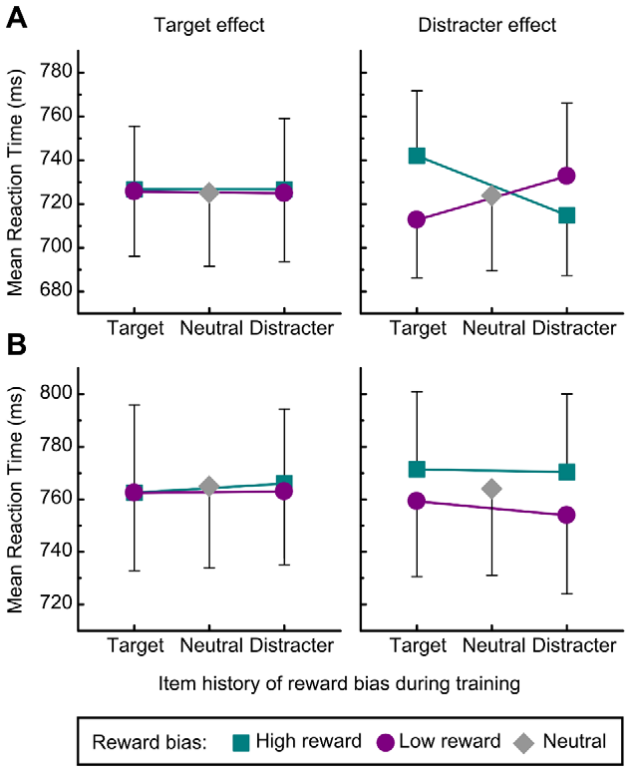
Behavioral results. a) Results obtained in our previous research, showing that the effect of reward bias depended on the attentional role of each stimulus during biased reward delivery ([28] Exp. 1). b) Results obtained in the present Experiment, showing a generalized effect of reward bias, unaffected by the associated attentional roles. Note that the y-axes scales in a) and b) slightly differ. In all plots error bars denote SEM.

The fact that VSA mechanisms are shaped and refined through learning may be crucial for exerting the most efficient guidance of human behavior. In a recent set of studies we have highlighted that the attentional priority of visual stimuli not only depends on the frequency with which they have been encountered in the past, but more interestingly on the *consequences* that followed processing of such stimuli [27], [28]. Using a typical VSA task – where subjects were asked to respond to a target while ignoring a distracter – each correct response received a monetary reward, that could have a low or a high value (i.e., 1 or 10 eurocents, respectively). While all stimuli were used the same number of times as targets or distracters, the probability of receiving a high or low reward in return for a correct behavioral response was biased, depending on which item had been shown as target or distracter in the stimulus array during the rewarded trial (Figure 1). While rewards were independent of actual performance (e.g., speed or overall accuracy rate), our participants were deceitfully told instead that reward amounts did depend on their performance, so that of all correct responses the more efficient would gain high rewards, and the poor ones low rewards. Following this manipulation some striking effects emerged, so that, for instance, targets whose *selection* had been more successful (i.e., more frequently followed by high reward) became favorite targets of future selections, and conversely, distracters whose *inhibition* had been more successful became easier to filter out when displayed as distracters on future occasions [28] (e.g., see Figure 2a).

The relationship between attentional performance and reward has been further explored in other recent studies [29]–[34], which confirmed and extended the evidence for robust attentional biases determined by controlled reward delivery. In visual search, highly rewarded targets led to faster and more accurate performance and stronger inter-trial priming effects [29]–[33]. Moreover, stimuli more frequently associated with high rewards became resilient to the attentional blink phenomenon [34].

Other studies, however, have demonstrated that the biased delivery of rewards can affect processing of any visual stimulus that was displayed close in time to the reward signal, even in the absence of conscious processing of reward-related cues [35], and even with minimal or null attentional requirements of the given task [36], [37]. As a consequence, stimuli associated with rewarding events became favorite objects [35], were more easily discriminated [36], [37], and were more vigorously represented in visual cortical processing areas [36].

Interestingly, while all these studies addressed the effects of biased stimulus-reward associations on performance, the type of instructions given to the participants relatively to the nature of rewards has received less consideration, as if it were irrelevant for the effects to emerge. In some cases subjects were fully debriefed that some stimuli would yield higher rewards in turn for a correct behavioral response [32], [34]–[36], while in others the relationship between stimuli and outcome probability was concealed, and subjects were deceitfully encouraged to maximize the earned rewards *as if* the earned amount could be determined by their performance [8], [27]–[31]. Finally, in some cases rewards were delivered while subjects were engaged in a passive viewing task, with no behavioral or attentional requirements [36]–[37].

Given the latter evidence, one might suspect that *any* effect of reward on perceptual or attentional processing might rely on a passive association between the experimental stimuli and the more-or-less rewarding events that accompany them. Thus, irrespectively of any link between one's own performance and the high or low rewards received afterwards, similar perceptual and/or attentional adjustments should emerge following sufficient exposure to a biased reward schedule.

Here we aim to directly explore the origin and nature of the reward-related effects found in our previous work [28]. For the present purposes we replicated exactly the conditions in Experiment 1 of our previous study [28], but this time we informed subjects that rewards were *random*, *lottery-like* wins, and that these random gains would sum up to constitute their compensation for participating to the experiment. Two contrasting predictions can be made. On the one hand, one might expect to replicate the exact same pattern of results that we obtained in our original study, regardless of the different instructions given to the participants. On the other hand, one might expect that the effects we obtained previously would weaken or even vanish once deception of participants is removed, indicating that the original results depended crucially on active monitoring of performance and outcome by our subjects. To our surprise, neither prediction was confirmed, as reported below.

## Materials and Methods

### Participants, Stimuli and Apparatus

Sixteen naïve participants took part in the study (8 males, mean age 23). The stimuli were 16 outlined nonsense shapes (2°×2°), as previously used [27], [28]. Stimulus displays were presented on a 17-inch CRT monitor in a quiet and dimly lit room, at a viewing distance of 90 cm. All participants read and signed an informed consent form prior to participating to the experiments. Throughout the research project leading to this publication, the rights of the experimental subjects were protected and the applicable guidelines concerning the use of human subjects for the purposes of research were followed. The study was approved by the ethics committee of the Department of Neurological, Neuropsychological, Morphological and Movement Sciences of the University of Verona.

### Task and Reward Schedules

Task and reward assignments replicated exactly those used by Della Libera and Chelazzi [28], Exp. 1 (Figure 1). Subjects participated in four experimental sessions, each lasting about one hour and comprising 960 trials. The first three sessions were regarded as *training*, and took place on consecutive days. The fourth and last session was regarded as a *test*, and took place 5 days after the last training session. Throughout all experimental sessions (both training and test), subjects performed in a task where nonsense figure stimuli were to be conveniently selected and ignored. On each trial, two overlapping shapes, one in red and one in green, appeared at 3° of eccentricity on the left of fixation, while a single black shape was displayed at the same eccentricity on the right. Subjects were to make a same/different judgment between one of the shapes on the left and the black shape on the right, by pressing one of two keys with the index or middle finger of the right hand. Each trial started with a green or red colored square (0.5°×0.5°) displayed centrally for 400 ms, and signaling which of the two shapes on the left was relevant during the trial. Six hundred ms after cue offset, the stimulus display was presented for 3 s or until the subject's response. Only during the training sessions, correct responses were followed by reward, which could be high (€0.10) or low (€0.01), and the amount was shown on the monitor for 600 ms. No reward was instead delivered during the test session. In all cases errors were followed by a high pitch tone for 800 ms, and the new trial started after a 1 s inter-trial interval.

Stimulus displays were designed so that each of the 16 shapes appeared as target, distracter, or comparison item the same number of times. Target and comparison item were identical in 50% of cases, requiring a “same” response. In the remaining trials, which required a “different” response, the comparison item differed from both target and distracter.

The only difference between this experiment and Exp. 1 in our prior study [28] consisted in the instructions given to subjects prior to their participation in the training sessions. Here participants were informed that reward values did not depend on performance level, provided that their response was correct, and could be regarded as random, lottery-like wins awarded to each correct response. The reward value delivered on each trial was in fact predetermined and balanced across all experimental conditions, being high or low with the same overall probability (50%). However, the schedule of reward assignment was crucially biased so that at the end of training the sixteen total stimuli could be divided into different categories depending on the proportion of high versus low rewards received in trials where they served as target or distracter (see Figure 1b). Two shapes were followed by a high reward in 80% of trials where they had been a target (T+); two other shapes were followed by a high reward in 80% of trials where they had been a distracter (D+). Two shapes were followed by a high reward in 20% of trials where they had been a target (T−); and finally two other shapes were followed by a high reward in 20% of trials where they had been a distracter (D−). Importantly, when these eight shapes were displayed in the role that was not associated with a bias in the reward schedule (e.g., when a T+ item was presented as a distracter), they led to a high or low reward with equal probability. The remaining eight shapes were used as neutral fillers, and they led to high or low reward in 50% of cases both when displayed as target and distracter. In order to avoid any possible confound given by the fixed association of individual shapes to each of the experimental categories, the same sixteen shapes were sorted differently across categories for each participant.

## Results

We focused our analysis on performance during the test session, including reaction times (RT) of correct responses and error rates (ER). Since none of the differences in ER across conditions reached statistical significance, they are not reported. Average ER was 3%.

For RT analysis, trials were first grouped according to the type of shape used as the target in the current test trial, and data were submitted to a ANOVA with reward bias (80% high/20% low or 20% high/80% low) and item history (bias applied when target or distracter during training) as main factors. Similarly to what we had found in our previous work [28], neither of the main effects nor the interaction between the two reached statistical significance (reward bias: *F*(1,15) = 0.033, n.s.; item history: *F*(1,15) = 0.059, n.s.; reward bias×item history: *F*(1,15) = 0.094, n.s.) (Figure 2b, left panel).

A second ANOVA was then conducted according to the type of shape used as the distracter in the trial. The ANOVA with reward bias (80% high/20% low or 20% high/80% low) and item history (target or distracter) as main factors revealed a reliably significant effect of reward bias (*F*(1,15) = 5.564, *p*<0.04, η^2^_p_ = 0.271), while both the effect of item history and the interaction between the two factors were far from significance (item history: *F*(1,15) = 0.25, n.s.; reward bias×item history: *F*(1,15) = 0.08, n.s.) (Figure 2b, right panel).

Therefore, unlike what we had found in the earlier study, here the attentional bias determined by reward delivery emerged as a main effect for all stimuli that overall had been more frequently followed by high vs. low reward, regardless of the role (target or distracter) played during training when differential rewards were delivered. Stimuli more often associated with high rewards during training were now more difficult to ignore with respect to stimuli more often associated with low rewards.

Given that the present experiment and Exp. 1 in [28] are identical, except for the different instructions given to participants about rewards, we carried out supplementary between-subjects analyses in order to directly compare the results from the two experiments. Two separate ANOVAs, one relative to target effects and the other relative to distracter effects, were carried out on data from the two experiments, with deception (present-absent) as the between-subjects factor. In fact, while in the old experiment subjects were deceived that rewards depended on their performance, here rewards were told to be given on a random basis (provided that the behavioral response was correct). The other factors were reward bias (80% high/20% low or 20% high/80% low) and item history (target or distracter), as in the previous analyses.

Results from the between-subjects ANOVA relative to the target effect revealed no significant main effect or interaction with the between-subjects factor, reasserting that the reward related manipulations applied during training do not emerge in the form of target-selection biases in the current task (but see [28], Exp. 2).

The second ANOVA, relative to distracter effects, showed instead an overall significant effect of reward bias (*F*(1,30) = 5.969, *p*<0.03, η^2^_p_ = 0.166), indicating a small but reliable global disadvantage in responding on trials with a highly rewarded distracter (749.7 ms, 80% high/20% low vs. 739.7 ms, 20% high/80% low). The effect of item history was non significant, nor was its interaction with the between-subject factor deception, while the interaction between item history and reward bias was significant both overall (*F*(1,30) = 5.041, *p*<0.04, η^2^_p_ = 0.144) and in its second order interaction with deception (*F*(1,30) = 7.306, *p*<.02, η^2^_p_ = 0.196). The latter, higher-order effect reveals that the interaction between reward bias and item history was present only in the deceived group (*F*(1,15) = 17.868, *p*<0.001, η^2^_p_ = 0.544; post-hoc *t* tests: T+ vs. T−, *t*(15) = 3.715, *p*<0.002, T+ vs. D+, *t*(15) = 3.738, *p*<0.002; D− vs. T−, *t*(15) = 2.169, *p*<0.05; D− vs. D+, *t*(15) = 2.281, *p*<0.04) (Figure 2a, right panel). The striking difference between data in the right panels of Figure 2, a and b, underscores the crucial influence of instructions given to the participants in the present as opposed to our prior experiment.

## Discussion

The present results extend considerably our knowledge on the mechanisms underlying reward-related learning effects in visual selective attention. The attentional processing of distracters, which was crucial for task performance, proved to be particularly sensitive to the delivery of biased rewards during training. Following extensive training with biased reward delivery, distracter rejection showed robust adjustments that could still be observed after a 5 days delay. Strikingly, such adjustments are not insensitive to the informative value of reward signals. If, and only if, performance is thought to be instrumental in leading to high or low gains, then the adjustments will affect the specific attentional computations engaged during execution of the reward-worthy behavioral response [28]. Hence, for instance, inhibition will become more efficient for stimuli that in the past have been ignored with highly successful outcomes (i.e., D+ items), while it will remain relatively poor for those that have been ignored the same number of times, but with less favorable results (i.e., D−). Inhibition is also impaired when dealing with items that have been highly rewarded when selected as targets (i.e., T+) relatively to items whose selection has more frequently led to lower gains (i.e., T−) [28]. Instead, when performance, and therefore the cognitive operations underlying it, is thought to bear no causal relationships with the following rewarding events, significant adjustments are observed which *do not* keep track of the specific attentional computations that were determinant for behavioral responses. In this case attentional inhibition is only affected by the overall frequency with which each stimulus had been followed by high or low reward during training. As a consequence, all items (targets and distracters) more frequently followed by high rewards (all “+” items) became more difficult to ignore during the test session. Incidentally, it should be noted that given our 80-20 reward imbalance, active only for stimuli shown in one attentional role (target or distracter), and the 50-50 reward probability when the same stimuli were shown in the alternative role, the overall effect of reward bias reflects a rather small overall imbalance in reward levels, namely 65-35. Should this imbalance be increased, most likely the effects shown in Fig. 2b (right) would grow larger.

Looking at these results from the viewpoint of the more traditional associative learning literature, the delivery of monetary rewards seems to affect VSA in ways that are similar to those available for the modification of overt behavior (e.g., [38]–[41]). Thus, when performance is viewed as determinant in leading to rewards, learning modulates the specific attentional processes that enabled it (i.e., target selection and distracter inhibition) and a form of instrumental attentional learning is observed (i.e., [42]). In contrast, when rewards are non related to performance, as in the present study, then stimuli become associated with the rewards that have been coupled with them, regardless of the attentional operations underlying response selection – within Pavlovian associative learning (e.g., [43]), a phenomenon similar to evaluative conditioning (e.g., [44], [45]).

In general, human associative learning can be strongly influenced by both the instructions delivered to participants [46]–[48], and the participants' beliefs on the contingencies between performance and outcome [48], [49]. These beliefs may also affect the degree to which the learning-related modifications may generalize and involve different levels of stimulus representations (for a discussion see [50], pp. 204–207). Interestingly, Dickinson et al. [49] demonstrated that when participants are initially exposed to an experimental context in which outcomes are determined by external causes, they fail to acquire instrumental learning when outcomes are later rendered contingent on their own performance. We might speculate that in our present study the explicit instruction that rewards were delivered randomly similarly *blocked* (i.e., [51], [52]) instrumental learning for the behaviorally relevant contingencies.

According to the notion of Pavlovian-to-instrumental transfer (e.g., [53]), instrumental learning emerges following two consecutive phases: initially, a perceptual stimulus is associated with a contiguous reward in a Pavlovian fashion. Subsequently, subjects learn to act in response to this stimulus in order to evoke the associated reward. If this were also the case for the attentional learning that we have induced, then we might conjecture that when rewards are viewed as unrelated to performance only the first – automatic – association is formed, and only the overall contingencies between stimulus representations and rewards will be learned. If instead rewards are thought to depend on performance, then the process develops completely and learning modulates not just a generic stimulus representation, but specifically the attentional weighing (or prioritization) process acting on this representation (i.e., selected as a target or inhibited as a distracter).

Taken altogether, these findings reveal that rewards can determine long-term changes in visual selective attention by means of at least two distinct mechanisms. One such mechanism is triggered by active monitoring of performance and outcome, and comes into play when rewards signify feedback on behavioral efficiency. The other mechanism instead detects the co-occurrence between processing of certain objects in the environment (whether attended or ignored) and the rewarding value of the events that accompany them, and reflects the establishment of direct, passive associations between the two.

Both types of learning effect may be accompanied by lasting changes in the neural representation of reward-associated stimuli in visual cortex [26], [35], [36], [54]. In addition, areas involved in attentional processing and control [55] and several nodes of the reward processing system [56], [57], including the striatum (e.g., [54]), the orbitofrontal cortex (e.g., [54]), and sectors of the anterior cingulate cortex (e.g., [30]), might similarly participate in both our learning protocols. Furthermore, recent evidence suggests that specific subcortical structures, such as portions of the striatum [58]–[60], might be crucially recruited only when rewards are perceived to depend on performance, giving rise to action-dependent instrumental learning. It would then be particularly interesting to explore whether training with such different types of attentional learning protocols recruits specific and dissociable patterns of brain activity, both at the cortical and subcortical level, highlighting the contribution of specific brain structures to distinct forms of reward-mediated attentional learning.

## References

[1] Duncan J (2006). EPS Mid-Career Award 2004: Brain mechanisms of attention.. Q J Exp Psychol.

[2] Egeth HE, Yantis S (1997). Visual attention: Control, representation, and time course.. Annu Rev Psychol.

[3] Pashler HE (1999). The Psychology of Attention.

[4] Reynolds JH, Chelazzi L (2004). Attentional modulation of visual processing.. Annu Rev Neurosci.

[5] Chun MM, Turk-Browne NB (2007). Interactions between attention and memory.. Curr Opin Neurobiol.

[6] Chun MM, Golomb JD, Turk-Browne NB (2011). A taxonomy of external and internal attention.. Annu Rev Psychol.

[7] Desimone R (1996). Neural mechanisms for visual memory and their role in attention.. Proc Natl Acad Sci U S A.

[8] Kristjànsson A, Campana G (2010). Where perception meets memory: A review of repetition priming in visual search tasks.. Atten Percept Psychophys.

[9] Logan GD (2002). An instance theory of attention and memory.. Psychol Rev.

[10] Chun MM (2000). Contextual cueing of visual attention.. Trends Cogn Sci.

[11] Chun MM, Jiang Y (1998). Contextual cueing: implicit learning and memory of visual context guides spatial attention.. Cogn Psychol.

[12] Chun MM, Jiang Y (1999). Top-down attentional guidance based on implicit learning of visual covariation.. Psychol Sci.

[13] Ciaramitaro VM, Cameron EL, Glimcher PW (2001). Stimulus probability directs spatial attention: an enhancement of sensitivity in humans and monkeys.. Vision Res.

[14] Dixon ML, Ruppel J, Pratt J, De Rosa E (2009). Learning to ignore: Acquisition of sustained attentional suppression.. Psychon Bull Rev.

[15] Geng JJ, Behrmann M (2002). Probability cuing of target location facilitates visual search implicitly in normal participants and patients with hemispatial neglect.. Psychol Sci.

[16] Hoffman J, Kunde W (1999). Location-specific target expectancies in visual search.. J Exp Psychol Hum Percept Perform.

[17] Jiang Y, Chun MM (2001). Selective attention modulates implicit learning.. Q J Exp Psychol A.

[18] Kelley TA, Yantis S (2009). Learning to attend: Effects of practice on information selection.. J Vis.

[19] Kelley TA, Yantis S (2010). Neural correlates of learning to attend.. Front Hum Neurosci.

[20] Kyllingsbæk S, Schneider WX, Bundesen C (2001). Automatic attraction of attention to former targets in visual displays of letters.. Percept Psychophys.

[21] Miller J (1988). Components of the location probability effect in visual search tasks.. J Exp Psychol Hum Percept Perform.

[22] Shiffrin RM, Schneider WX (1977). Controlled and automatic human information processing: II. Perceptual learning, automatic attending, and a general theory.. Psychol Rev.

[23] Tang Y-Y, Posner MI (2009). Attention training and attention state training.. Trends Cogn Sci.

[24] Tseng C-H, Gobell JL, Sperling G (2004). Long-lasting sensitization to a given colour after visual search.. Nature.

[25] Vidnyànszky Z, Sohn W (2005). Learning to suppress task-irrelevant visual stimuli with attention.. Vision Res.

[26] Jagadeesh B, Chelazzi L, Mishkin M, Desimone R (2001). Learning increases stimulus salience in anterior inferior temporal cortex of the macaque.. J Neurophysiol.

[27] Della Libera C, Chelazzi L (2006). Visual selective attention and the effects of monetary rewards.. Psychol Sci.

[28] Della Libera C, Chelazzi L (2009). Learning to attend and to ignore is a matter of gains and losses.. Psychol Sci.

[29] Hickey C, Chelazzi L, Theeuwes J (2010). Reward primes target selection in visual search but not suppression of distractors.. Vis Cogn.

[30] Hickey C, Chelazzi L, Theeuwes J (2010). Reward changes salience in human vision via the anterior cingulate.. J Neurosci.

[31] Hickey C, Chelazzi L, Theeuwes J (2010). Reward guides vision when it's your thing: Trait reward-seeking in reward-mediated visual priming.. PLoS One.

[32] Kiss M, Driver J, Eimer M (2009). Reward priority of visual target singletons modulates event-related potential signatures of attentional selection.. Psychol Sci.

[33] Kristjànsson A, Sigurjònsdòttir O, Driver J (2010). Fortune and reversals of fortune in visual search: Reward contingencies for pop-out targets affect search efficiency and target repetition effects.. Atten Percept Psychophys.

[34] Raymond JE, O'Brien JL (2009). Selective visual attention and motivation: the consequences of value learning in an attentional blink task.. Psychol Sci.

[35] Pessiglione M, Petrovic P, Daunizeau J, Palminteri S, Dolan RJ (2008). Subliminal instrumental conditioning demonstrated in the human brain.. Neuron.

[36] Frankó E, Seitz AR, Vogels R (2010). Dissociable neural effects of long-term stimulus-reward pairing in macaque visual cortex.. J Cogn Neurosci.

[37] Seitz AR, Kim D, Watanabe T (2009). Rewards evoke learning of unconsciously processed visual stimuli in adult humans.. Neuron.

[38] Castro L, Wasserman EA (2010). Animal learning.. Wiley Interdiscip Rev Cogn Sci.

[39] Shanks DR (1993). Human instrumental learning: A critical review of data and theory.. Br J Psychol.

[40] Shanks DR (2007). Associationism and cognition: Human contingency learning at 25.. Q J Exp Psychol.

[41] Shanks DR (2010). Learning: From association to cognition.. Annu Rev Psychol.

[42] Dickinson A, Mackintosh NJ (1994). Instrumental conditioning.. Animal learning and cognition.

[43] Hall G, Mackintosh NJ (1994). Pavlovian conditioning. Laws of association.. Animal learning and cognition.

[44] De Houwer J, Baeyens F, Field AP (2005). Associative learning of likes and dislikes: Some current controversies and possible ways forward.. Cogn Emot.

[45] De Houwer J, Thomas S, Baeyens F (2001). Associative learning of likes and dislikes: A review of 25 years of research on human evaluative conditioning.. Psychol Bull.

[46] Baron A, Kaufman A, Stauber KA (1969). Effects of instructions and reinforcement-feedback on human operant behavior maintained by fixed-interval reinforcement.. J Exp Anal Behav.

[47] Hayes SC, Brownstein AJ, Haas JR, Greenway DE (1986). Instructions, multiple schedules, and extinction: Distinguishing rule-governed from schedule-controlled behavior.. J Exp Anal Behav.

[48] Rosenfarb IS, Newland MC, Brannon SE, Howey DS (1992). Effects of self-generated rules on the development of schedule-controlled behavior.. J Exp Anal Behav.

[49] Dickinson A, Shanks DR, Evenden JL (1984). Judgment of act-outcome contingency: The role of selective attribution.. Q J Exp Psychol A.

[50] Baddeley AD (1990). Human Memory: theory and practice.

[51] Kamin LJ, Mackintosh NJ, Honig WK (1969). Selective association and conditioning.. Fundamental Issues in Instrumental Learning.

[52] Kruschke JK, Blair NJ (2000). Blocking and backward blocking involve learned inattention.. Psychon Bull Rev.

[53] Lovibond PF (1983). Facilitation of instrumental behavior by a pavlovian appetitive conditioned stimuls.. J Exp Psychol Anim Behav Process.

[54] Weil RS, Furl N, Ruff CC, Symmonds M, Flandin G (2010). Rewarding feedback after correct visual discriminations has both general and specific influences on visual cortex.. J Neurophysiol.

[55] Peck CJ, Jangraw DC, Suzuki M, Efem R, Gottlieb J (2009). Reward modulates attention independently of action value in posterior parietal cortex.. J Neurosci.

[56] O'Doherty JP (2004). Reward representations and reward-related learning in the human brain: insights from neuroimaging.. Curr Opin Neurobiol.

[57] Schultz W (2006). Behavioral theories and the neurophysiology of reward.. Annu Rev Psychol.

[58] Tricomi EM, Delgado MR, Fiez JA (2004). Modulation of caudate activity by action contingency.. Neuron.

[59] Bjork JM, Hommer DW (2007). Anticipating instrumentally obtained and passively-received rewards: A factorial fMRI investigation.. Behav Brain Res.

[60] Hakyemez HS, Dagher A, Smith SD, Zald DH (2008). Striatal dopamine transmission in healthy humans during a passive monetary reward task.. Neuroimage.

